# Prolonged resuscitation and systemic management of multi-organ dysfunction in a neonate with severe asphyxia: a case report

**DOI:** 10.3389/fped.2025.1592769

**Published:** 2025-09-15

**Authors:** Qingsong Wang, Jun Yin, Junru Wang, Zhihui Zhao, Taojun Du, Huimin Ou, Hongmei Cao, Hong Zhang, Haonan Cao, Wenlong Yue, Siguang Jiang, Tongyong Luo, Xianmin Wang

**Affiliations:** ^1^ Department of Pediatrics, Ultrasound, Obstetrics and Gynecology, Rehabilitation, Anesthesiology, Intensive Care Unit, West China Hospital Sichuan University Jintang Hospital, Jintang First People’s Hospital, Chengdu, Sichuan, China; ^2^ Department of Neonatology, Sichuan Provincial Women’s and Children’s Hospital/The Affiliated Women’s and Children’s Hospital of Chengdu Medical College, Chengdu, Sichuan, China; ^3^ Pediatric Cardiology Center, Sichuan Provincial Women’s and Children’s Hospital/The Affiliated Women’s and Children’s Hospital of Chengdu Medical College, Chengdu, Sichuan, China

**Keywords:** neonatal asphyxia, prolonged resuscitation, multi-organ dysfunction syndrome (MODS), systemic management, case report

## Abstract

Severe neonatal asphyxia can lead to multiple organ dysfunction syndrome (MODS) and increase mortality and disability risks. This case report describes the successful resuscitation and management of a neonate who experienced 15 min of severe asphyxia. The male infant, born at 36 weeks’ gestation via emergency cesarean section, had extremely low Apgar scores (1 at 1, 5, and 10 min). He underwent 15 min of resuscitation, including airway clearance, endotracheal intubation, positive pressure ventilation, chest compressions, and multiple administrations of epinephrine. Post-resuscitation, he exhibited severe dysfunction in multiple organ systems. The infant received comprehensive treatment, including invasive mechanical ventilation, continuous renal replacement therapy (CRRT) for acute kidney injury, therapeutic hypothermia for neuroprotection, surgical treatment for necrotizing enterocolitis with perforation, and specialized nutritional support. His condition significantly improved, with resolution of MODS, and he was discharged with weight gain and good feeding tolerance. Neurological assessments at discharge showed no significant abnormalities; however, long-term follow-up is ongoing to monitor for potential neurodevelopmental outcomes. This case highlights the importance of timely resuscitation and meticulous systemic management in achieving a favorable prognosis for neonates with severe asphyxia and MODS. The successful collaboration of a multidisciplinary team played a key role in the neonate's recovery.

## 1 Introduction

Perinatal asphyxia is a leading cause of neonatal death and long-term neurological sequelae, with its pathogenesis involving multiple organ dysfunction syndrome (MODS) triggered by hypoxic-ischemic reperfusion injury. Although the incidence is relatively low, it poses a significant threat to life once it occurs, especially in cases requiring prolonged resuscitation, and is a major factor in neonatal mortality and infantile disability (1). This case report details the successful survival of a neonate who underwent prolonged resuscitation after experiencing 15 min of severe asphyxia. Following resuscitation, the infant exhibited severe dysfunction in multiple systems, including respiratory, circulatory, digestive, hematologic, and neurological systems. Through close collaboration of a multidisciplinary team and active systemic treatment, the infant ultimately recovered and was discharged from the hospital. This detailed case report aims to provide further insights into the management of neonatal asphyxia requiring prolonged resuscitation, particularly in the management of MODS, as well as how to improve the success rate of treatment through multidisciplinary cooperation. This case also highlights the potential impact of underlying metabolic disorders (such as tyrosinemia) in the context of neonatal asphyxia.

## 2 Case presentation

### 2.1 Maternal medical history

The patient's menstrual cycle was regular, and pregnancy was confirmed at 30 days of amenorrhea with typical early pregnancy symptoms. Prenatal care was initiated at 17 weeks of gestation, with irregular prenatal examinations. Key findings included mild mitral and tricuspid regurgitation, which did not affect daily activities. Non-invasive DNA testing yielded a low-risk result. At 20 weeks of gestation, a monochorionic diamniotic twin pregnancy was confirmed. Fetal echocardiography showed mild tricuspid regurgitation in Fetus A, while Fetus B exhibited no significant abnormalities. The patient reported biweekly examinations at a local hospital, with no reported abnormalities. An oral glucose tolerance test (OGTT) was not performed during the pregnancy. At 31 weeks of gestation, the second systematic ultrasound showed increased placental thickness and breech position of Fetus B. Fetal echocardiography revealed mild tricuspid regurgitation and a narrow aortic arch diameter in Fetus B. From the fourth month of pregnancy until delivery, the patient consistently felt fetal movements. No special treatment was administered throughout the pregnancy.

### 2.2 Neonatal birth history

The male infant was delivered at 36 weeks’ gestation via emergency cesarean section, weighing 1970 grams. The mother had a complicated pregnancy, including fetal distress, transverse lie, monochorionic diamniotic twin pregnancy (with the other fetus being stillborn), marginal umbilical cord insertion, moderate anemia, thrombocytopenia, and placental abruption (Grade 3). The amniotic fluid was clear, with no nuchal cord.

At birth, the infant had no cry, flaccid limbs, pale skin, and no spontaneous breathing. Auscultation revealed a heart rate of approximately 30–40 beats per minute. Immediate resuscitation was initiated, including airway clearance, endotracheal intubation (completed within 1 min of birth), positive pressure ventilation, chest compressions, and multiple administrations of epinephrine (1:10,000 epinephrine 1 ml via endotracheal tube 3 times, and 0.3 ml via umbilical vein 5 times). Volume expansion with normal saline was performed twice. After 15 min of resuscitation, the heart rate increased to 120 beats per minute, with oxygen saturation maintained above 90%. The infant was then transferred to the neonatal intensive care unit (NICU) under endotracheal intubation and positive pressure ventilation.

The Apgar scores were 1 at 1, 5, and 10 min (heart rate 1 point). At 15 min, the score was 6 (heart rate 2 points, respiration 1 point, color 1 point, muscle tone 1 point, stimulation 1 point). At 20 min, the score was 7 (heart rate 2 points, respiration 1 point, color 1 point, muscle tone 1 point, stimulation 1 point).

### 2.3 Clinical presentation upon admission to the NICU

The infant was admitted to the NICU at 44 min of age, with the chief complaint of “44 min post-asphyxia resuscitation”. Physical examination under endotracheal intubation and positive pressure ventilation with a bag mask revealed: T 35 ℃, P 130 beats per minute, R 36 breaths per minute, SpO2 80%, blood pressure 43/11 mmHg, poor response, pale complexion, no jaundice, shallow and weak breathing, flat anterior fontanelle, symmetrical breath sounds in both lungs, regular heart rhythm, weak heart sounds, no murmurs heard, soft abdomen, liver not palpable, spleen not palpable, decreased muscle tone in all four limbs, absence of rooting, sucking, grasp, and Moro reflexes.

### 2.4 Ancillary tests

Umbilical cord blood gas analysis revealed severe acidosis (pH 6.68, BE −26.6 mmol/L) with hypercapnia (PCO_2_ 105.7 mmHg) and hyperlactatemia (13.26 mmol/L); admission blood gas analysis showed acidosis (pH 6.89, BE −26.7 mmol/L, lactate 14.71 mmol/L). Hematologic testing indicated anemia (Hb 110 g/L), with marked coagulopathy (PT 106.7 s, APTT 126.8 s, FIB 0.19 g/L, D-dimer 111.6 µg/ml). Cardiac enzymes were elevated (TnI 0.191 ng/ml, CK 629 U/L, CK-MB 200 U/L), while liver function tests showed hypoproteinemia (TP 18.9 g/L) and elevated AST (269 U/L); renal function was normal (Crea 63 µmol/L). Echocardiography identified a patent ductus arteriosus (PDA) with right-to-left shunting, mild pulmonary hypertension (pulmonary artery systolic pressure, PASP 36 mmHg). Cranial ultrasound suggested subependymal hemorrhage (increased echogenicity), and lung ultrasound revealed dense B-lines bilaterally. Abdominal imaging showed renal pelvis separation and enhanced parenchymal echogenicity, while chest x-ray demonstrated coarse lung markings. Amplitude-integrated EEG (aEEG) showed discontinuous normal voltage with an immature sleep-wake cycle. Hemodynamic monitoring (ICON device) revealed elevated intrathoracic fluid (66 ohms^1^) and myocardial contractility (192.9), with a cardiac index of 4.6 L/min/m^2^². Cranial MRI at 2 weeks confirmed cerebellar hemorrhagic foci, and ophthalmic screening at 4 weeks detected stage 1 retinopathy in zone III bilaterally. Blood and sputum cultures were negative, and the neonatal behavioral neurological assessment (NBNA) score at discharge was 37.

## 3 Discussion

### 3.1 Multiple organ dysfunction and treatment strategies in severe neonatal asphyxia

Neonatal asphyxia is a respiratory and circulatory dysfunction caused by hypoxia in the fetus during intrauterine or delivery processes. Infants may present with apnea or an inability to establish regular breathing after birth, accompanied by abnormal gas exchange. If this condition persists, it can further develop into severe hypoxemia and hypercapnia, often complicated by metabolic acidosis (2). As an independent etiological factor for multi-organ damage, the incidence of perinatal asphyxia has been reported as 3.29% in a multicenter study (3), and it can cause irreversible damage to multiple organs. The incidence of early organ damage in perinatal asphyxia is 75.30%, and in neonates with severe asphyxia, it reaches 96.67%. The severity of organ damage typically ranks as follows: brain, lung, heart, kidney, liver, and gastrointestinal tract (4). Currently, the incidence of multi-organ damage in perinatal asphyxia in China ranges from 37.70% to 79.30%, with mortality rates both domestically and internationally ranging from 4.80% to 4.87% (5).

Pathophysiologically, neonatal asphyxia can induce systemic hypoxia, leading to redistribution of blood flow to prioritize perfusion of the heart, brain, and adrenal glands, while other organs or systems suffer ischemic injury due to inadequate perfusion (6). In this case, the infant experienced severe asphyxia for 15 min, reaching the limit of the body's self-regulation mechanism. Failure of compensatory mechanisms in critical organs (brain, heart, adrenal glands) was associated with progressive dysfunction of the nervous, circulatory, respiratory, digestive, and blood systems. Upon admission, the infant presented with neonatal hypoxic-ischemic encephalopathy, neonatal shock, acute kidney injury, severe pneumonia, myocardial damage, hepatic dysfunction, and upper gastrointestinal bleeding, with supportive examinations revealing corresponding abnormalities. These findings are consistent with the organ damage typically observed in severe neonatal asphyxia.

However, this case is unique in that the most severe damage was observed in the lungs, kidneys, and gastrointestinal tract, whereas the brain, heart, and liver exhibited relatively mild injury. Specifically, the brain did not present with clinical symptoms associated with severe hypoxic-ischemic encephalopathy, with minimal intracranial hemorrhage and mild-moderate abnormalities on electroencephalogram; the heart did not experience cardiogenic shock, with normal cardiac function test results; and liver enzyme tests did not indicate significant abnormalities. In contrast, severe pulmonary injury necessitated invasive mechanical ventilation; acute kidney injury required continuous renal replacement therapy (CRRT); and gastrointestinal involvement included upper gastrointestinal bleeding at admission, which progressed to necrotizing enterocolitis (NEC) with intestinal perforation during the course of illness. Additionally, coagulation function was significantly impaired, with manifestations of disseminated intravascular coagulation leading to the occurrence of shock.

**Figure 1:**
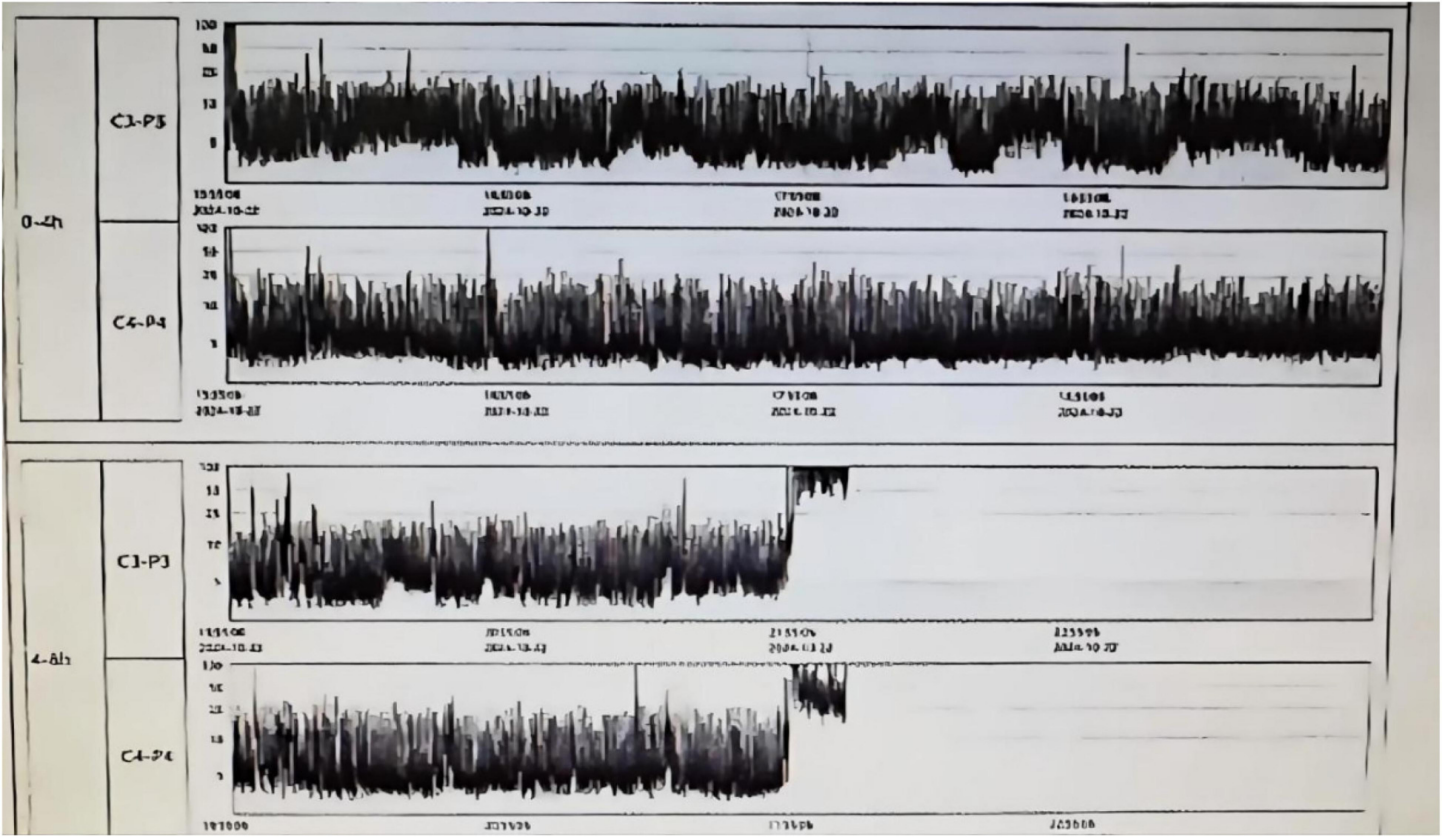
Neonatal brain function monitoring report on the second day of life, showing moderately abnormal electroencephalographic activity.

The mechanism of brain injury involves mitochondrial dysfunction, oxidative stress, and neuroinflammation caused by hypoxia-ischemia, leading to neuronal apoptosis and abnormal cortical migration (7). Hypoxic-ischemic encephalopathy (HIE) is classified into mild, moderate, and severe stages (Sarnat staging), with severe HIE being associated with mortality and long-term cognitive impairments (8). The neuroprotective treatment strategy for this patient included initiating therapeutic hypothermia within 5 h of birth, with a target temperature of 33.5–34.5°C for brain protection, and head immobilization to reduce further injury. However, due to hemostasis difficulties at the puncture site, hypothermia treatment was discontinued after 21 h. At 22 h of age, the infant exhibited increased muscle tone, and an EEG showed moderately abnormal electroencephalographic activity (suggesting possible seizure episodes, see Figure 1), prompting treatment with phenobarbital for 3 days to control epilepsy and protect neurological function.

Given the limitations of echocardiography in dynamically monitoring key parameters such as cardiac output (CO) and systemic vascular resistance (SVR) during therapeutic hypothermia (TH) for neonatal HIE (9), we employed non-invasive cardiac output monitoring technology (e.g., OSYPKA MEDICAL GMbH ICON device) to assess hemodynamics and guide cardiovascular support therapy (10–12). This approach allows for continuous monitoring and trend analysis, which is crucial for identifying disease progression and adjusting vasoactive drugs (13). The choice of monitoring methods should be individualized and integrated into a goal-directed hemodynamic management strategy (14, 15).

The mechanism of cardiac injury involves adenosine triphosphate (ATP) depletion, calcium overload, and the release of cardiac troponin I (cTnI) in myocardial cells due to hypoxia, leading to circulatory system disorders characterized by anuria, severe metabolic acidosis, unstable blood pressure, and acute kidney injury (AKI), manifesting as neonatal shock and renal failure (16, 17). After admission, volume expansion and infusion of vasoactive drugs (dopamine, norepinephrine, epinephrine) were administered to stabilize blood pressure and correct acidosis. CRRT in CHDF mode was initiated 12 h and 28 min after birth, concurrently with therapeutic hypothermia. A detailed summary of the arterial blood gas analysis from admission to CRRT treatment is provided in Table 1. Elevated cardiac enzyme spectrum and troponin levels indicated myocardial damage, and fructose-1,6-diphosphate was administered for myocardial nutrition to promote cardiac function recovery.

**Table 1:** Arterial blood Gas analysis from admission to CRRT treatment.

Time Point	pH	PCO₂ (mmHg)	PO₂ (mmHg)	BE (mmol/L)	${\mathrm{HCO}}_{3}^{-}$ (mmol/L)	LAC (mmol/L)
Umbilical cord blood gas	6.683	105.7	24.4	−26.6	7.2	13.26
October 21, 10:29	6.885	34.8	90	−26.7	6.5	14.7
October 21, 13:07	7.39	26.5	81.0	−7.8	15.8	20.2
October 21, 17:59	7.261	24.8	73.9	−7.9	18.2	21.5
October 22, 01:08	7.282	19.8	90	−17.6	16.5	24.3
Before CRRT	7.346	33.6	36.2	−7.7	18.9	3.63
After CRRT	7.397	39.3	33.8	−1.2	23.6	4.65
October 24, 22:22	7.415	35.2	50.8	−2.2	22.5	4.93

Prolonged hypoxia and acidosis in this infant led to dysfunction of pulmonary artery endothelial cells and smooth muscle cells, causing pulmonary vasoconstriction and worsening pulmonary microcirculation, resulting in respiratory failure and pulmonary hypertension. Upon admission, invasive mechanical ventilation was initiated using High-Frequency Oscillatory Ventilation (HFO). After 15 h, the ventilation mode was adjusted to synchronized intermittent mandatory ventilation (SIMV) due to improved oxygenation. Invasive mechanical ventilation continued for 62 h until the infant's consciousness returned and breathing stabilized, at which point non-invasive ventilation was initiated. Non-invasive ventilation was maintained for 90 h before the infant was weaned off the ventilator due to good spontaneous breathing capacity. The infant had non-persistent pulmonary hypertension with no hypoxemia under mechanical ventilation, thus inhaled nitric oxide therapy was not administered. Initially, cefotaxime was used for anti-infection treatment due to severe asphyxia and pulmonary infection at birth. On the second day, considering the infant's prematurity and critical condition, the treatment was escalated to piperacillin/tazobactam. Subsequently, due to the development of necrotizing enterocolitis with perforation, meropenem was administered for 3 days after consultation with the pharmacy department, followed by a step-down to piperacillin/tazobactam for another 5 days.

**Figure 2:**
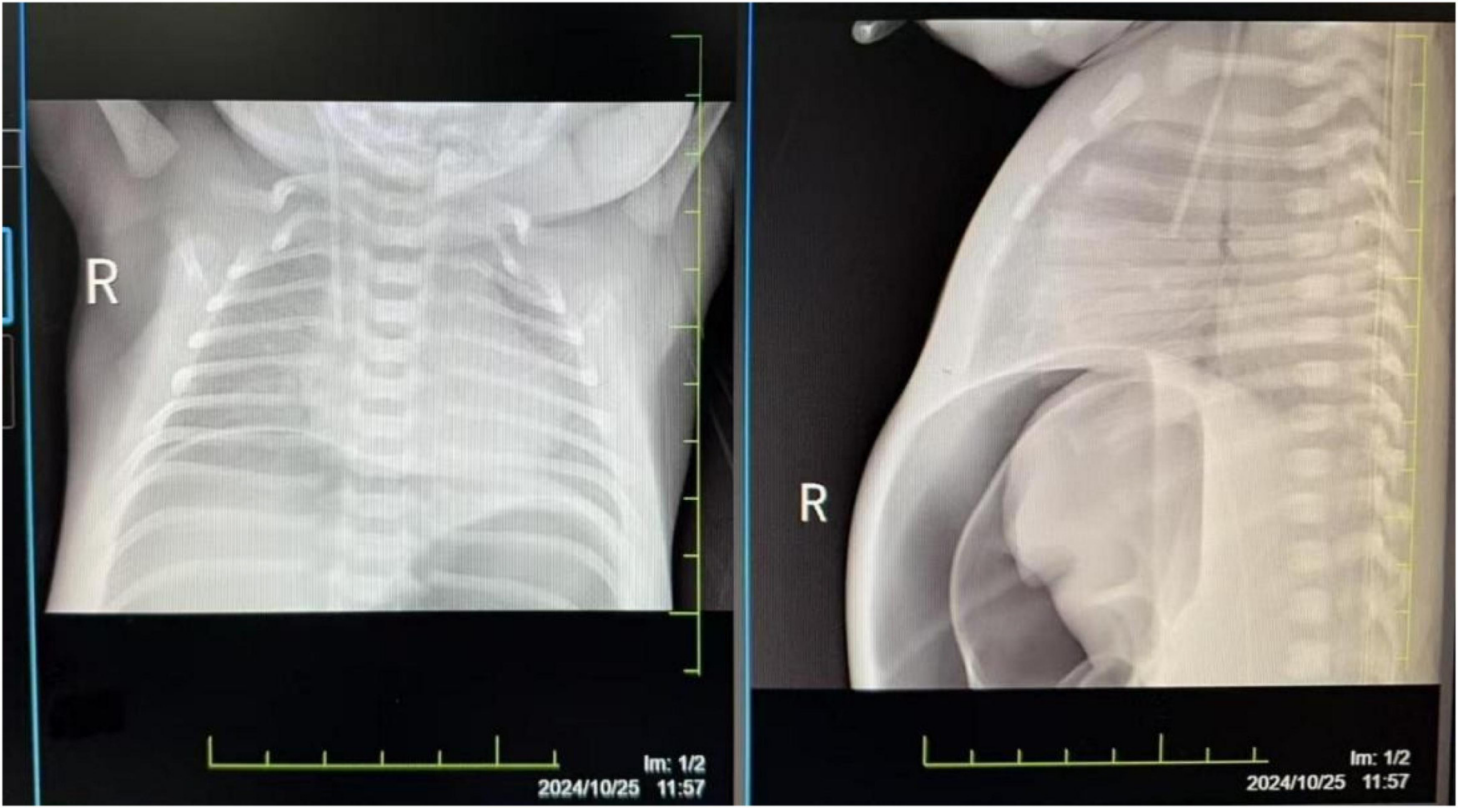
Abdominal radiograph taken on the fourth day after birth, showing pneumoperitoneum.

**Figure 3:**
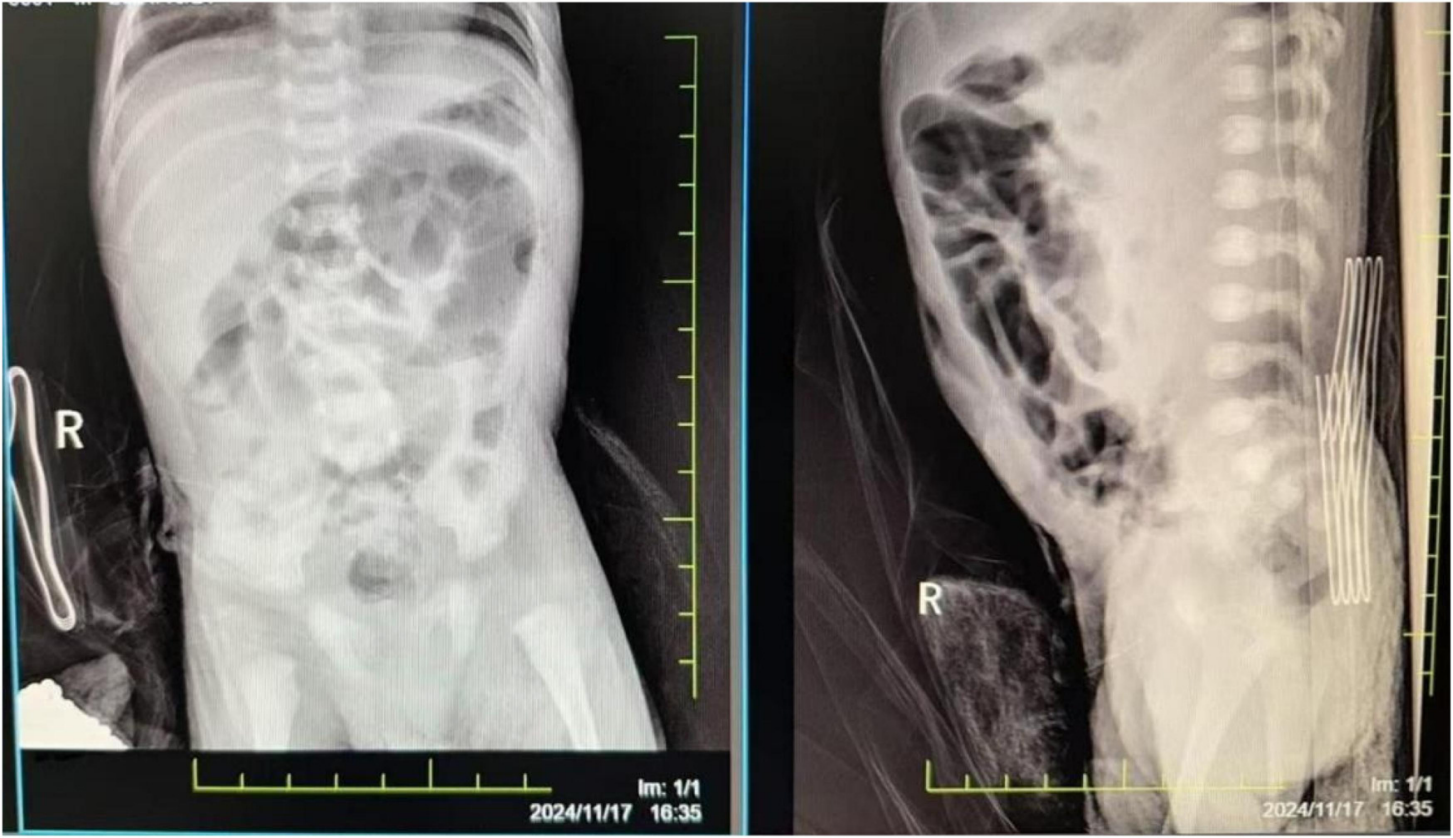
The abdominal radiograph on postoperative day 21 suggests recurrent necrotizing enterocolitis.

The damage to the gastrointestinal system is due to the redistribution of blood flow initiated to protect more critical organs, with significant reduction in intestinal blood supply. This leads to edema, increased permeability, ulceration, necrosis, and bleeding of the gastrointestinal mucosa, gastrointestinal dysfunction, and reperfusion damage, resulting in particularly severe intestinal injury. Severe asphyxia-related gastrointestinal damage is positively correlated with the incidence of multiple organ dysfunction (2). Although protective measures, including fasting, were implemented for the gastrointestinal system after admission, the infant developed necrotizing enterocolitis with perforation on the fourth day after birth (see Figure 2). Intraoperatively, extensive congestion and edema of the small intestine from 10 cm to 40 cm from the ileocecal valve were observed, covered with purulent exudate, with poor intestinal wall elasticity; intestinal wall rupture, edema of the mesenteric root, and numerous gas bubbles were visible. An ileal resection, intestinal anastomosis + ileostomy (Santulli procedure) + abdominal drainage were performed. After confirming the return of intestinal function, breastfeeding was initiated on the fourth day after surgery, gradually increasing milk volume to full enteral nutrition. Subsequently, due to the development of cholestasis, the feeding was changed to an extensively hydrolyzed milk formula after consultation with a gastroenterologist. On the 21st day after surgery, the infant again had bloody stools, and an abdominal radiograph suggested recurrent necrotizing enterocolitis (see Figure 3). Immediate fasting was initiated, and parenteral nutrition support was started, along with cefoperazone/sulbactam for anti-infection treatment.

In this case, it was also found that the infant did not gain weight when primarily fed enterally from the second week of life, experienced feeding difficulties, slight somnolence, and persistent metabolic acidosis. Although daily sodium bicarbonate was administered to correct acidosis, the effect was not satisfactory. Subsequent tandem mass spectrometry of blood and urine for inherited metabolic diseases revealed tyrosine levels of 321.68 µmol/L and 619.47 µmol/L, respectively. Two blood and urine tandem mass spectrometry tests suggested tyrosinemia. Although subsequent genetic testing did not identify any pathogenic/likely pathogenic gene variants or copy number variations (CNVs) that could clearly explain the clinical findings, the infant's metabolic acidosis was corrected after feeding with a low phenylalanine, low tyrosine formula combined with a 20% extensively hydrolyzed protein formula.

Hemodynamic redistribution after asphyxia can also lead to insufficient hepatic perfusion, causing liver damage, metabolic disturbances in hepatocytes (such as hypoglycemia and lactate accumulation), and cholestasis (18). A multicenter study in China reported a liver damage incidence rate of only 7.8% (1). In 2016, a multicenter study in China revised the diagnostic criteria for liver damage in perinatal asphyxia to include alanine aminotransferase (ALT) levels > 80 U/L within 1 week of birth (19). However, in this case, only on day 28 did the liver function test show ALT at 92 U/L, while all other tests did not exceed this value. The initial liver function test after birth showed ALT at 21 U/L. On day 28, liver function showed ALT at 92 U/L, with total bile acid (TBA) at 57.9 µmol/L, leading to a diagnosis of cholestasis. Ademetionine disulfonate, reduced glutathione, and ursodeoxycholic acid were administered for hepatoprotection and choleretic therapy.

Severe asphyxia had a significant impact on the hematologic system, resulting in recurrent moderate to severe anemia, thrombocytopenia, and coagulation dysfunction. During the treatment process, multiple transfusions of red cells, platelets, plasma, and cryoprecipitate were required. The initial complete blood count showed moderate anemia (hemoglobin, HGB 110 g/L), which was corrected by transfusing 30 ml of leukoreduced suspended red cells.Concurrently, abnormal coagulation function [prolonged prothrombin time (PT), reduced fibrinogen] was addressed with fresh frozen plasma to replenish coagulation factors. Subsequently, the infant experienced recurrent hypoproteinemia, moderate to severe anemia, coagulation dysfunction, and thrombocytopenia. Consequently, albumin, red cells (including leukoreduced suspended red cells and washed red cells), fresh frozen plasma, cryoprecipitate, and irradiated single-donor platelets were transfused on multiple occasions to maintain hemodynamic stability, improve oxygen-carrying capacity, correct coagulation abnormalities, and prevent bleeding risks.

A study by domestic scholars on 3,123 infants at high risk of pregnancy or experiencing perinatal asphyxia within 72 h after birth revealed an incidence rate of retinal hemorrhage of 18%, with perinatal asphyxia being identified as the leading cause of retinal hemorrhage in high-risk infants (20). Additionally, studies have shown that the severity of asphyxia in neonates in the NICU is positively correlated with the severity of retinal hemorrhage, with an overall retinal hemorrhage rate reaching 28.29%. Among these neonates with retinal hemorrhage, the proportions of mild (Grade I), moderate (Grade II), and severe (Grade III) hemorrhages were 37.75%, 18.63%, and 43.63%, respectively, and 4 cases of vitreous hemorrhage were observed (21, 22). In this case, ophthalmic examination revealed stage 1 retinopathy of prematurity (ROP) in both eyes, with no specific treatment given at present, and follow-up observation in the ophthalmology outpatient clinic.

Maintaining internal environmental stability and electrolyte balance was crucial throughout the entire clinical course. To correct metabolic acidosis, multiple infusions of sodium bicarbonate were administered. Based on laboratory test results, calcium and magnesium ions were promptly supplemented to manage electrolyte disturbances such as hypocalcemia and hypomagnesemia, thereby helping to preserve neuromuscular and cardiac function. A detailed timeline of the major clinical events, including the management of metabolic acidosis and electrolyte disturbances, is provided in Table 2.

**Table 2:** Clinical timeline of the case.

Time Point	Event
October 21, 2024, 09:26	Birth (36 weeks’ gestation, emergency cesarean section). Immediate initiation of prolonged resuscitation (15 min) including airway clearance, endotracheal intubation, positive pressure ventilation, chest compressions, and multiple administrations of epinephrine.
October 21, 2024, 10:10	Transfer to NICU under endotracheal intubation and positive pressure ventilation with a bag mask.
October 21, 2024, 10:10	Continuous monitoring of vital signs initiated, and the airway kept patent. Start of high-frequency oscillatory ventilation (HFOV) with initial settings: FiO₂ 100%, MAP 17 cmH₂O, oscillation pressure 22 cmH₂O, frequency 10 Hz, while administering 1.4% sodium bicarbonate for acid correction and volume expansion.
October 21, 2024, 11:30	Start of vasoactive drug infusion (dopamine, norepinephrine, epinephrine) for hypotension and poor tissue perfusion.
October 21, 2024, 14:20	Initiation of therapeutic hypothermia for neuroprotection, target temperature 33.5–34.5 °C.
October 21, 2024, 21:34	Initiation of continuous renal replacement therapy (CRRT) due to acute kidney injury (AKI).
October 22, 2024, 01:00	Blood gas analysis shows significant improvement (pH 7.523, PaCO₂ 18.5 mmHg, PaO₂ 90 mmHg, SaO₂ 99.8%, BE −7.9 mmol/L, lactate 17.84 mmol/L). Transition to synchronized intermittent mandatory ventilation (SIMV) with settings: FiO₂ 30%, PEEP 6 cmH₂O, PIP 18 cmH₂O, RR 45 breaths per minute.
October 22, 2024, 11:56	Therapeutic hypothermia discontinued due to difficulty in hemostasis at puncture site.
October 23, 2024, 07:56	Increased muscle tone, phenobarbital administered for seizure prophylaxis.
October 24, 2024 15:27	Transition to non-invasive positive pressure ventilation (NIPPV) with settings: FiO₂ 25%, PEEP 6 cmH₂O, PIP 15 cmH₂O, RR 35 breaths per minute, Ti 0.38 s.
October 25, 2024 14:00	Onset of necrotizing enterocolitis (NEC) with intestinal perforation; Emergency laparotomy (ileal resection, intestinal anastomosis, ileostomy).
October 28, 2024 09:07	Discontinuation of mechanical ventilation.
November 8, 2024, 10:00	Initiation of acid correction and alkali supplementation, refractory metabolic acidosis noted.
November 15, 2024, 10:32	Diagnosis of inherited metabolic disorder (Tyrosinemia Type 1?).
November 19, 2024, 22:00	Recurrence of necrotizing enterocolitis (NEC).
November 30, 2024, 12:31	Initiation of tyrosine formula feeding.
December 4, 2024, 12:23	Improvement of refractory metabolic acidosis.
December 5, 2024, 15:28	Initiation of low phenylalanine, low tyrosine formula combined with 20% extensively hydrolyzed protein formula.
December 11, 2024, 22:43	Refractory metabolic acidosis corrected, alkali supplementation discontinued.
December 16, 2024, 15:26	Discharged from the hospital.

### 3.2 Differential diagnosis

The infant presented with multi-system dysfunction, necessitating thorough differential diagnosis to ensure accurate treatment. Neonatal hypoglycemia was ruled out due to normal serial glucose levels and absence of typical symptoms. Neonatal sepsis was unlikely given negative blood cultures and lack of systemic signs such as fever. Although the infant had congenital heart disease (patent ductus arteriosus, possible patent foramen ovale or atrial septal defect) and signs of pulmonary hypertension, these conditions were not severe, as the infant had no persistent cyanosis or respiratory distress, and maintained normal oxygen saturation under mechanical ventilation. Therefore, severe congenital heart disease and pulmonary hypertension were excluded.

Late tyrosinemia-like findings prompted a systematic assessment of congenital metabolic diseases. Initial tandem mass spectrometry showed elevated tyrosine, suggesting tyrosinemia. Despite negative genetic tests, metabolic profile and symptoms (acidosis, poor growth, feeding issues) supported tyrosinemia, further confirmed by lab findings like persistent acidosis and high lactate. Other metabolic disorders (organic acidemias, fatty acid oxidation defects, amino acid disorders) were excluded via negative tests and normal profiles. Metabolic specialists and genetic counselors confirmed tyrosinemia and advised parents. Management: low phenylalanine/tyrosine + 20% hydrolyzed protein formula, with regular follow-up for monitoring and treatment adjustment.

### 3.3 Discharge status and follow-up recommendations

After 56 days of recovery, the infant was discharged, showing good responsiveness and feeding tolerance. Fed with a low phenylalanine/tyrosine + 20% hydrolyzed protein formula, the infant's weight rose from 1,970 g at birth to 2,750 g at discharge, with a daily milk intake of 505 ml and 80 g yellow stool output the day before discharge. Physical exam showed stable vitals, slight jaundice, regular heart rhythm, clear lungs, soft abdomen, normal muscle tone, and intact primitive reflexes. EEG and NBNA score (37) were normal, with no neurological sequelae found, but close monitoring for seizures or other abnormalities is needed. Post-discharge, the infant should be followed up in the high-risk infant clinic for growth and neurological development, with timely rehabilitation referral if necessary. An echocardiogram should be done 3–6 months post-discharge, with pediatric cardiology follow-up, and nutrition clinic visits to adjust the feeding regimen.

## 4 Limitations of the case report

While this case report provides detailed insights into the management of severe neonatal asphyxia and MODS, it is important to acknowledge its limitations. The findings are based on a single case, which limits the generalizability of the results. The absence of a control group and the lack of long-term follow-up data mean that the long-term outcomes and potential sequelae of the interventions described cannot be fully assessed. Additionally, the genetic testing did not identify pathogenic variants associated with tyrosinemia, which introduces some uncertainty regarding the definitive diagnosis. Future studies with larger cohorts and longer follow-up periods are needed to validate these findings and better understand the implications of metabolic disorders in neonatal asphyxia.

## 5 Conclusion

This case report provides a detailed description of a neonate with severe asphyxia who developed multiple organ dysfunction and was successfully resuscitated and treated through precise diagnosis, individualized treatment plans, and multidisciplinary collaboration. This case offers valuable reference experience for clinical work in dealing with similar complex cases and highlights the potential impact of special metabolic diseases (tyrosinemia) in multiple organ dysfunction following neonatal asphyxia.

## Data Availability

The original data presented in this study are available within the article and its Supplementary Material. Further inquiries may be directed to the corresponding author.
